# Secondary infection with *Streptococcus suis *serotype 7 increases the virulence of highly pathogenic porcine reproductive and respiratory syndrome virus in pigs

**DOI:** 10.1186/1743-422X-7-184

**Published:** 2010-08-09

**Authors:** Min Xu, Shujie Wang, Linxi Li, Liancheng Lei, Yonggang Liu, Wenda Shi, Jiabin Wu, Liqin Li, Fulong Rong, Mingming Xu, Guangli Sun, Hua Xiang, Xuehui Cai

**Affiliations:** 1 Division of Swine Infectious Diseases, National Key Laboratory of Veterinary Biotechnology, Harbin Veterinary Research Institute, CAAS, Harbin 150001, China; 2 Veterinary Public Health Laboratory of Guangdong, Veterinary Medicine Institute, Guangdong Academy of Agricultural Sciences, Guangzhou 510640, China; 3 College of Animal Science and Veterinary Medicine, Jinlin University, Jilin 130062, China; 4 College of Veterinary Medicine, Northeast Agricultural University, Harbin 150030, China

## Abstract

**Background:**

Porcine reproductive and respiratory syndrome virus (PRRSV) and *Streptococcus suis *are common pathogens in pigs. In samples collected during the porcine high fever syndrome (PHFS) outbreak in many parts of China, PRRSV and *S. suis *serotype 7 (SS7) have always been isolated together. To determine whether PRRSV-SS7 coinfection was the cause of the PHFS outbreak, we evaluated the pathogenicity of PRRSV and/or SS7 in a pig model of single and mixed infection.

**Results:**

Respiratory disease, diarrhea, and anorexia were observed in all infected pigs. Signs of central nervous system (CNS) disease were observed in the highly pathogenic PRRSV (HP-PRRSV)-infected pigs (4/12) and the coinfected pigs (8/10); however, the symptoms of the coinfected pigs were clearly more severe than those of the HP-PRRSV-infected pigs. The mortality rate was significantly higher in the coinfected pigs (8/10) than in the HP-PRRSV- (2/12) and SS7-infected pigs (0/10). The deceased pigs of the coinfected group had symptoms typical of PHFS, such as high fever, anorexia, and red coloration of the ears and the body. The isolation rates of HP-PRRSV and SS7 were higher and the lesion severity was greater in the coinfected pigs than in monoinfected pigs.

**Conclusion:**

HP-PRRSV infection increased susceptibility to SS7 infection, and coinfection of HP-PRRSV with SS7 significantly increased the pathogenicity of SS7 to pigs.

## Background

Porcine reproductive and respiratory syndrome (PRRS) is a threat to the swine industry, and has spread globally to almost every country involved in pig farming, with a few exceptions like Sweden, Switzerland, New Zealand, and Australia [1-4]. PRRS mainly affects pigs and sows; it mainly causes premature delivery, miscarriage, stillbirth, and mummies in sows, and severe pneumonia, edema, and conjunctivitis in pigs. Previous studies have shown that coinfection of PRRSV and bacteria, such as *Streptococcus suis*, aggravates PRRS [5]. It has been confirmed that PRRSV-infected pigs are more susceptible to *S. suis *serotype 2 (SS2) infection, and the mortality rate of coinfected pigs is significantly higher than that of PRRSV infected pigs. While the clinical symptoms of both PRRSV infected and coinfected pigs were similar, the incidence of fever in coinfected pigs was higher than that in monoinfected pigs [6].

Since 2006, an unprecedented epidemic of porcine high fever syndrome (PHFS) has spread through the Chinese swine industry, resulting in the culling of an estimated 20 million pigs annually. This disease is characterized by high fever, high incidence rate, and high mortality rate [7]. After extensive epidemiological investigations, highly pathogenic PRRSV (HP-PRRSV) was first isolated from China; many reports suggested that an *NSP2 *and *GP5 *gene deletion mutant of PRRSV was the causative agent of the PHFS epidemic [7-10]. Since SS7 has been isolated from pigs exhibiting clinical symptoms of PHFS, some researchers also believe that PHFS is caused by coinfection of HP-PRRSV and *S. suis*. SS7 is more prevalent than other *S. suis *serotypes in several provinces of China [11,12], and SS7 exacerbates PHFS when accompanied by PRRSV infection. Other researchers have suggested that SS7 is less virulent than SS2 and cannot induce serious effects. We established a porcine model of single and mixed infection using veterinary clinical isolates of PRRSV strain HuN4 and SS7 strain WC0711 isolated from China, based on the protocol for coinfection model establishment proposed by Harms et al. [13]. Our aim was to characterize the interaction of SS7 with HP-PRRSV and to elucidate the causes of PHFS epidemic.

## Results

### Clinical Observation

The pigs in HP-PRRSV-infected group and coinfected group had rectal temperatures > 40.5°C for more duration than control pigs and SS7-infected pigs. Among the coinfected pigs, 8 died of acute illness and the remaining 2 survived. The mortality and morbidity rates of pigs in all groups are summarized in Table 1. The severity of respiratory disease in HP-PRRSV-infected and coinfected pigs was significantly higher (P < 0.05) than in control or SS7 monoinfected pigs. The HP-PRRSV-infected and coinfected pigs with respiratory disease had cough and sneezing in the early phase and asthma and abdominal breathing thereafter. The SS7-infected pigs exhibited mild and moderate cough and sneezing symptoms in the early phase, but they recovered within 2 or 3 days. The HP-PRRSV-infected (4/12) and coinfected pigs (8/10) showed signs of central nervous system (CNS) disease, including muscle tremors, head tilt, nystagmus, and posterior limb paralysis. Only 1 pig in the coinfected group exhibited joint swelling. These signs of CNS disease were not observed in SS7-infected pigs. Unlike SS7-infected pigs, all pigs in the HP-PRRSV-infected and coinfected groups exhibited athrepsia. Further, conjunctivitis was observed in HP-PRRSV-infected pigs and coinfected pigs and diarrhea was observed in HP-PRRSV-infected pigs (severe), SS7-infected pigs (temporary), and coinfected pigs (severe). The relevant clinical data are shown in Table 2.

**Table 1:** Mortality and morbidity rates of each group

**Group no**. (n)	Designation	Pigs Deaths*		Mortality	Morbidity
				
		Number	DPI		
1(8)	Control	0		0%^a^	0%^a^
					
2(12)	PRRSV	1	13	16.7%^b^	100%^b^
		1	21		
					
3(10)	SS7	0		0%^a^	70%^b^
					
4(10) **	PRRSV+SS7	1	10	80%^c^	100%^b^
		2	12		
		3	13		
		2	15		

*: Number of pigs and days post inoculation (DPI) when they were euthanized or died due to infection.

**: Two pigs were necropsied on day 7 (when the pigs were not inoculated with SS7).

a, b, c Values with different superscripts are significantly different (P < 0.05) as determined by Fisher's exact test.

**Table 2:** Respiratory disease scores and clinical signs

Group no.(n)	Designation	Respiratory disease scores	CNS disease	Diarrhea	Athrepsy	Bacteremia	Viremia
1(8)	Control	0.0 ± 0.0^a^	0/8^a^	0/8^a^	0/8^a^	0/8^a^	0/8^a^
2(12)	PRRSV	1.8 ± 0.3^b^	4/12^b^	5/12^b^	11/12^b^		12/12^b^
3(10)	SS7	1.0 ± 0.2^c^	0/10^a^	7/10^b^	0/10^a^	0/10^a^	
4(12)	PRRSV+SS7	2.1 ± 0.4^b^	0/2,8/10^c^	0/2,7/10^b^	0/2,10/10^b^	10/10^b^	12/12^b^

a, b, c, Values with different superscripts are significantly different (P < 0.05) as determined by Fisher's exact test.

### Macroscopic and microscopic lesions

**Figure 1:**
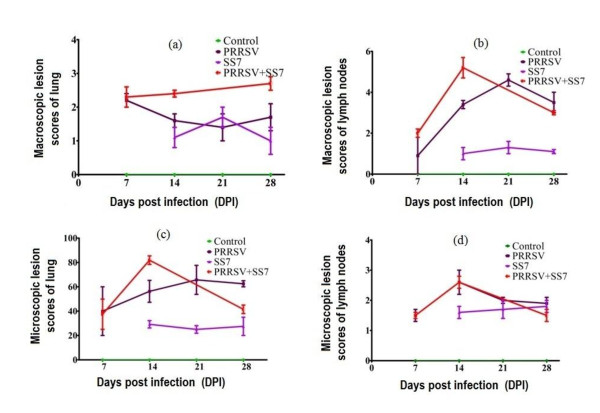
**Mean value (standard deviation) of lesion scores**. (a) Scores of macroscopic lung lesion, (b) Scores of macroscopic lymph node lesion, (c) Scores of microscopic lung lesion, and (d) Scores of microscopic lymph node lesion at different times after inoculation.

**Figure 2:**
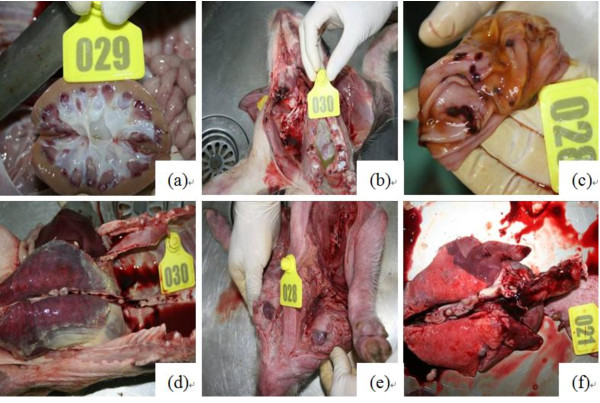
**Macroscopic lesions in the coinfected group**. (a) Kidney and pelvic hemorrhage; (b) Trichocardia and hydropericardium; (c) Stomach hemorrhage; (d) Bacterial infection in the thoracic cavity; (e) Lymph node intumescentia and hemorrhage; (f) Lung consolidation.

No macroscopic lesions were observed in the control pigs. The pigs in HP-PRRSV-infected and coinfected groups developed macroscopic brain lesions mainly due to meningitis, meningeal congestion and hemorrhage, swallowed gyri, and brain tissue colliquation. The lung lesions showed multifocal distribution. The affected lung parenchyma appeared mottled, tan, and rubbery, and failed to collapse. Several pigs in the coinfected group had pleuritis or purulent airway exudates. The severity of the lung lesions in SS7-infected pigs was lower than that in HP-PRRSV-infected and coinfected pigs. Pigs in all the 3 experimental groups showed lymph node lesions, including intumescentia and hemorrhage. The scores of lung and lymph nodes were evaluated in detail at necropsy, and are summarized in Figure 1. Kidney lesions in HP-PRRSV-infected and coinfected pigs included punctiform hemorrhage in the cortex, inner hemorrhage, shrunken or disappeared substantia medullaris, and change of color to cinnamon. Macroscopic lesions were also detected in other organs in HP-PRRSV-infected and coinfected pigs; these pigs had grayish-white spots in the liver, and trichocardia, hydrops pericardial, tonsillitis, hemorrhage of the stomach fundus, and spleen infarcts. The SS7-infected pigs had macroscopic lesions mainly in the lungs and lymph nodes with few lesions in other organs. Peritonitis was observed in SS7-infected pigs (2/10) and coinfected pigs (4/10). There was no evidence of arthritis in any of the pigs. The representative images of the macroscopic lesions are presented in Figure 2.

**Figure 3:**
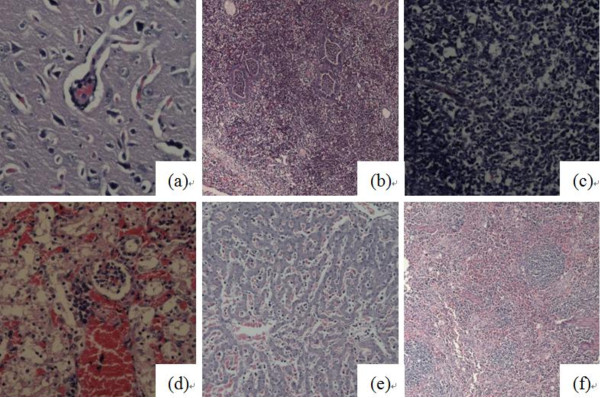
**Microscopic lesions in the coinfected group**. (a) Perivascular cuffs in brain; (b) Suppurative pneumonia and purulent bronchitis; (c) Lymphocyte necrosis and disintegration in submaxillary lymph nodes; (d) Severe congestion of kidney and renal tubular necrosis and collapse; (e) Moderate sinusoidal congestion with a small number of lymphoid cells and macrophage infiltration in the lung; (f) Lymphoid cell necrosis and collapse around the ellipsoid arterioles and in the white pulp and red pulp of the spleen.

The control pigs showed no pathological lesions. The pathological lung lesions in HP-PRRSV-infected pigs showed the following characteristics: widened interval of alveolar and mild interstitial pneumonia on day 7; interstitial pneumonia associated with purulent lesions and small vessel thrombosis on day 14; suppurative pneumonia and purulent bronchitis on day 21; and localized interstitial pneumonia on day 28. The pathological lung lesions in SS7-infected pigs showed the following characteristics: widened alveolar septa with neutrophil suppurative lesions and small vessel thrombosis on day 14 (7 days after inoculation); widened alveolar septa accompanied by a small number of lymphoid cells, eosinophil infiltration with some fibrin deposition, and mild interstitial pneumonia with neutrophil infiltration on day 21; and small amount of lymphocytic infiltration, fibroplasia, small vessel thrombosis and extensive localized interstitial lymphoid cell infiltration around the bronchi on day 28. The pathological lung lesions in all of the 8 deceased pigs (death occurred between days 10 and 15) exhibited suppurative pneumonia, purulent bronchitis, thrombosis, blood stasis, and widened alveolar septa with fibrin deposition. The severity of the lesions in the deceased pigs was significantly higher (P < 0.01) than that in HP-PRRSV-infected pigs on day 14. The 2 surviving pigs showed localized interstitial pneumonia on day 28. Their lymph nodes exhibited marked lymphocyte necrosis and disintegration, which was sometimes combined with plasma cell hyperplasia or eosinophil infiltration, elevated nodule volume, nodule cavitation, and paracortical hemorrhage. The lymph nodes of HP-PRRSV-infected and coinfected pigs showed a considerable degree of injury with no significant difference, but were obviously more serious (P < 0.01) than those in the SS7-infected pigs. The specific pathology scores of microscopic lung and lymph nodes are summarized in Figure 1. The pathological examination of the brain of PRRSV- and SS7-infected pigs mainly revealed small vessel thrombosis, mild edema, neuron liquefaction necrosis, and glial cell infiltration and perivascular cuffing in some pigs. However, serious perivascular cuffing and thrombosis were observed in the pathological specimens of coinfected pigs. The representative images of the microscopic lesions are presented in Figure 3.

### Bacteremia and viremia

Bacteremia was observed in all 10 pigs of the coinfected group, 2 of which showed persistent bacteremia during the experiment. No bacteremia was observed in SS7-infected pigs. At 3 days after HP-PRRSV infection, viremia was observed in all pigs of the HP-PRRSV-infected and coinfected groups, and 9 pigs had viremia even on day 1. Except 5 pigs, all pigs had viremia throughout the study.

### Organ-wise distribution of virus and bacteria

The HP-PRRSV strain HuN4 and SS7 strain WC0711 were not detected in the organs of the control pigs. The distribution status of HuN4 and WC0711 strains in 9 organs at different time points after inoculation is summarized in Tables 3 and 4. WC0711 strain detection rates in the 9 organs of the 8 deceased pigs in the coinfected group were significantly higher (P < 0.05) than those in SS7-infected pigs at all time points after inoculation, except in the liver and tonsils on day 28. HuN4 strain detection rates in the brains and stomachs of coinfected pigs were significantly higher (P < 0.05) than those in HP-PRRSV-infected pigs, while the detection rate in other organs was identical for both the groups. Secondary infection with SS7 improved the distribution of the strain HuN4. The strain HuN4 was detected in all the 9 examined organs in the 8 deceased pigs; the detection rate of HuN4 in the brain of coinfected pigs was up to 50%, which was much higher than that in the monoinfected groups.

**Table 3:** Distribution of HP-PRRSV in 9 organs at different times after inoculation

Designation	Day	brain	tonsil	Lymph node	heart	liver	spleen	lung	kidney	stomach
PRRSV	7	0/4	4/4	4/4	2/4	1/4	2/4	4/4	1/4	0/4
	13-14*	0/4	4/4	4/4	3/4	0/4	4/4	4/4	3/4	0/4
	21	0/3	2/3	3/3	2/3	1/3	0/3	3/3	1/3	0/3
	28	1/3	3/3	3/3	3/3	3/3	0/3	3/3	3/3	0/3
										
PRRSV+SS7	10-15**	4/8	8/8	8/8	7/8	5/8	6/8	8/8	7/8	2/8
	28	0/2	1/2	2/2	0/2	0/2	1/2	2/2	1/2	0/2

*: One pig died on day 13 and the remaining 3 pigs were necropsied on day 14.

**: Eight pigs in the coinfected group died due to acute illness between days 10 and 15 after WC0711 inoculation, and the remaining 2 were necropsied on day 28.

**Table 4:** Distribution of *Streptococcus suis *serotype 7 in 9 organs at different times after inoculation

Designation	day	brain	tonsil	Lymph node	heart	liver	spleen	lung	kidney	stomach
SSV	14	1/4	1/4	1/4	0/4	1/4	1/4	0/4	2/4	0/4
	21	0/3	1/3	0/3	0/3	1/3	0/3	0/3	1/3	0/3
	28	1/3	3/3	0/3	1/3	3/3	0/3	0/3	0/3	0/3
										
PRRSV+SS7	10-	7/8	7/8	5/8	5/8	5/8	7/8	6/8	5/8	5/8
	28	1/2	2/2	1/2	0/2	0/2	0/2	1/2	0/2	2/2

### PRRSV RNA quantification

**Figure 4:**
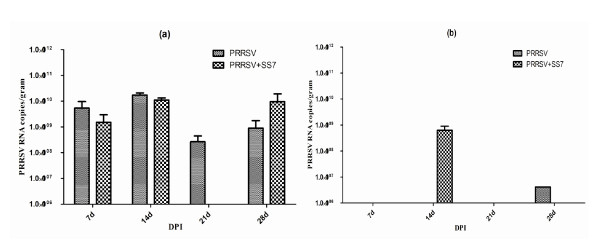
**PRRSV RNA**. (a) PRRSV RNA levels in the lungs of all pigs in HP-PRRSV-infected and coinfected groups either after necropsy or death. The data from the coinfected group at 14 days post-inoculation (DPI) is the mean value of 8 pigs of the coinfected group that died between 10 and 15 DPI; no pigs from the coinfected group were necropsied on 21 DPI. (b) The PRRSV RNA levels in the brains of HP-PRRSV-infected and coinfected pigs.

The control and SS7-infected pigs did not contain PRRSV RNA in the lungs and brain. However, all pigs in the HP-PRRSV-infected and coinfected groups contained PRRSV in the lungs, and 4 pigs in the coinfected group and 1 pig in HP-PRRSV-infected group contained PRRSV in the brain. The lungs of HP-PRRSV-infected and coinfected pigs contained similar amount of PRRSV RNA; however, the amount of PRRSV RNA in the brain of coinfected pigs was significantly higher (P < 0.01) than that in HP-PRRSV-infected pigs. The PRRSV levels in the lung and brain are shown in Figure 4.

## Discussion

HP-PRRSV has been a great threat to pigs since 2006 [7]. However, in this study, we found that PRRSV alone cannot cause extensive damage to the host. Pigs coinfected with PRRSV and SS7 showed symptoms similar to those characteristic of PHFS: high fever, high mortality and morbidity, severe respiratory disease, red coloration of the body, and blue colored ears. The SS7 strain WC0711, an isolate obtained from a PHFS endemic area, was weakly pathogenic in pigs; the severity of the clinical symptoms of the SS7-infected pigs was low and there were no fatalities. This result was consistent with the findings of previous reports regarding the weak virulence of SS7. The SS7 strain WC0711 is an attenuated strain; it can cause mild or moderate respiratory symptoms in pigs, but self-healing may start within a short duration [14]. However, the condition of pigs coinfected with HP-PRRSV (HuN4) and SS7 (WC0711) deteriorated rapidly, with deaths occurring continuously after day 10. The rectal temperature of the 8 deceased pigs of the coinfected group reached 41°C, and in 3 of the 8 pigs the temperature reached 41.5°C, and the number of neurologic symptoms in these pigs increased significantly. Secondary infection with the strain WC0711 increased the mortality of PRRSV-infected pigs from 16.7% to 80% and their progression to the acute stage. We suggest that even SS7, which is a weak virulent serotype, can cause significantly more damage when coinfected with HP-PRRSV. Our results were consistent with those of Feng et al., who reported that after uterine infection of PRRSV in sows, the pigs were markedly more susceptible to SS2 at birth [6].

Several reports have confirmed that the main characteristic of PRRSV is its damaging effects on the host immune system, which facilitates the invasion of other pathogens [5,15-17]. The immunosuppressive effects are most potent 1 week after infection with PRRSV [18]. The lymph node injury in coinfected pigs was more severe than that in PRRSV (HuN4)-infected pigs. Therefore, we speculated that HP-PRRSV and PRRSV have similar destructive effects on the immune system, and allow secondary infection by SS7, thereby further exacerbating the damage to the immune system. In addition, neurotoxicity is an important feature of PRRSV infection in pigs [19]. Brain injury caused by other pathogens is aggravated after coinfection with PRRSV [20]. In our study, CNS diseases did not develop in the SS7-infected pigs, but the incidence of CNS diseases was doubled in the coinfected pigs as compared to that in the HP-PRRSV-infected pigs. Therefore, coinfection of HP-PRRSV and SS7 may damage the nervous system or induce HP-PRRSV to aggravate nervous system injuries. This effect may be due to the fact that HP-PRRSV infection increases the permeability of the blood-brain barrier. SS7 caused bacteremia immediately after HP-PRRSV infection and rapidly spread to each organ, aggravating the pathological injury to the lungs, brain, and lymph nodes; the combined effect of coinfection markedly increased host mortality. Moreover, SS7 infection improved the distribution of the virus, thereby causing damage to more organs. Therefore, secondary infection with SS7 enhances the pathogenicity of HP-PRRSV in pigs.

## Conclusions

We have shown that the clinical manifestations of the coinfection model are similar to those observed during the "high fever" epidemic in China since 2006. The SS7 epidemic may have played a role in the occurrence and development of PRRS. The general level of importance of SS7 has always been less than SS2 because of the characteristic low virulence of the former. However, pigs coinfected with HP-PRRSV and SS7 exhibited serious clinical symptoms affecting multiple organs, such as the lungs, brain, and lymph nodes, which reduced the production and increased mortality, particularly in the case of newborn pigs. Therefore, it is necessary to control bacterial infection by adopting measure to prevent and control HP-PRRS infection. Further characterization of PHFS of pigs under laboratory and natural conditions will be necessary to understand the mechanisms underlying the enhanced susceptibility of HP-PRRSV-infected pigs to SS7.

## Methods

### Animals

We brought 42 crossbred early-weaned pigs of approximately 3 weeks of age to our research facility. The pigs were purchased from a herd determined to be free of PRRSV and SS7 based on regular serological testing. On the day of arrival, the pigs were randomly assigned to 4 groups: 8, control group; 12, HP-PRRSV-infected group and coinfected group; and 10, SS7-infected group. The care and treatment of pigs in our study were conducted in accordance with the guidelines on animal experimentation of Chinese Academy of Agricultural Sciences (CAAS).

### Experimental design and inoculations

Eight pigs served as uninoculated controls. The pigs in the HP-PRRSV-infected group and coinfected group were intranasally (IN) (2 ml) and intramuscularly (IM; 1 ml) inoculated with the representative strain of HP-PRRSV, namely, HuN4 on day 0 [21]. The pigs in the SS7-infected group and coinfected group were IN (2 ml) and IM inoculated with the SS7 strain WC0711, which was isolated from a sample of PHFS, on day 7. The experimental design is summarized in Table 5. The HP-PRRSV strain HuN4 isolated at passage 25 was used as the inoculum for virus challenge. The titer of the virus was calculated as 10^4.97 ^TCID50/2 ml. The WC0711 strain was isolated from a pure culture on sheep blood agar plates (BAPs) by overnight incubation at 37°C, and was subsequently cultured in Todd-Hewitt broth (Bacto, Liverpool, UK) with 5% horse serum for 12 h at 37°C. The culture was centrifuged at 3,000 rpm for 15 min and the bacteria were resuspended in phosphate buffered saline (PBS). The bacterial inoculum contained approximately 10^9 ^colony-forming units (CFU)/ml.

**Table 5:** Experimental design for coinfection and postmortem examination

Group no. (n)	Designation	Treatment at age (dpi)		Necropsy at age (dpi)			
		
		0 d	7 d	7 d	14 d	21 d	28 d
1(8)	Control			2	2	2	2
2(12)	HP-PRRSV	HuN4		2	4	3	3
3(10)	SS7		WC0711	0	4	3	3
4(12) *	HP-PRRSV+SS7	HuN4	WC0711	2	6	2	2

*: Eight pigs in the coinfected group died of acute illness between days 10 and 13, as shown in Table 1, after WC0711 inoculation, and the remaining 2 pigs were necropsied on day 28.

### Clinical observation

The pigs were monitored daily and scored for the severity of clinical respiratory disease by using a scoring system ranging from 0 (normal) to 6 (severe dyspnea and abdominal breathing) [22]. In addition, the pigs were observed daily for other clinical signs, including CNS disease, joint swelling, lameness, sneezing, and jaundice. The rectal temperature, muscle wasting, and behavioral changes, such as lethargy were recorded daily.

### Postmortem examination

We randomly selected 2 pigs each from the control group, HP-PRRSV-infected group, and coinfected group and performed necropsy on day 7 of the study. Then, 4 randomly selected pigs from all the 3 experimental groups and 2 from the control group were necropsied on day 14 and 21, respectively. The remaining pigs were necropsied on day 28. If the pigs died or became moribund before schedule, they were necropsied immediately. Organ samples (brain, tonsils, lymph nodes, heart, liver, spleen, lungs, kidneys, and stomach) were collected and analyzed for the presence of SS7 by polymerase chain reaction (PCR). Reverse transcriptase (RT)-PCR [5] and real-time PCR [23] for HP-PRRSV and immunofluorescent antibody (IFA) assays for both SS7 and HP-PRRSV [24] were performed. The distribution of virus and bacteria was investigated.

An estimated percentage of the lung affected by pneumonia was visibly determined for each pig based on a scoring system [22]. The sections of heart, liver, and kidney were evaluated for the presence of lymphohistiocytic inflammation and scored from 0 (none) to 3 (severe). Lymphoid tissues, including lymph nodes, tonsils, and spleen, were evaluated for lymphoid depletion ranging from 0 (normal) to 3 (severe) and histiocytic inflammation and replacement of follicles ranging from 0 (normal) to 3 (severe) [25]. The macroscopic and microscopic scores were evaluated by 4 different researchers.

### Bacteremia and viremia

To monitor bacteremia and viremia, blood was collected on days 0, 1, 3, 5, 7, 8, 10, 12, 15, 19, 23, and 28. Whole blood was cultured in Todd-Hewitt broth for the detection of SS7 by PCR. The presence of PRRSV in the sera was determined by RT-PCR using previously described methods [13]. DNA was extracted from whole blood using a TIANamp bacteria DNA kit (TIANGEN) and RNA was extracted from the sera using a QIAamp viral RNA mini kit (QIAGEN). The emergence and persistence times of bacteremia and viremia were recorded. The primers used for the detection of bacteremia were cps7a: 5'-AGTCTAACACGAAATAAGGC-3' and cps7b: 5'-GTCAAACACCCTGGATAGCCG-3'. The primers used for the detection of viremia were HP-PRRSVVU: 5'-GAGGGAAGGGGATTGCCAGCCAG-3' and HP-PRRSVVL: 5'-CTCACCCCCACACGGTCGCCCTAA-3'.

### Statistical analysis

Before data analysis, descriptive statistics were performed to assess the overall quality of the data. Continuous data were analyzed by one-way analysis of variance (ANOVA). If the results of one-way ANOVA showed significant difference (P < 0.05), pairwise testing using Tukey's adjustment was performed. Discrete data (macroscopic and microscopic lesions and clinical observations) were analyzed by the nonparametric Kruskal-Wallis one-way ANOVA. If the results of nonparametric ANOVA showed significant difference (P < 0.05), Wilcoxon tests were performed for pairwise analysis. Response feature analysis was performed to account for clinical observations. The results obtained after HP-PRRSV challenge were combined, and the averages for each pig and differences among groups were compared by nonparametric Kruskal-Wallis ANOVA. Fisher's exact test was used to evaluate the differences in the incidence of infection.

## Competing interests

The authors declare that they have no competing interests.

## Authors' contributions

Xu Min carried out the animal experiments, participated in the sequence alignment and drafted the manuscript. Wang Shujie and Liu Yonggang cultured the virus and bacteria. Shi Wenda, Li Liqin, and Wu Jiabin were responsible for the molecular genetic studies. Rong Fulong carried out the immunoassays. Xu Mingming participated in the sequence alignment. Sun Guanli, Li Linxi and Xiang Hua participated in the design of the study and performed the statistical analysis. Cai Xuehui and Lei Liancheng conceived of the study, participated in its design and coordination, and helped to draft the manuscript. All authors read and approved the final manuscript.
